# Development and characterization of a novel bulk-fill elastomeric temporary restorative composite

**DOI:** 10.1590/1678-7757-2018-0183

**Published:** 2018-12-10

**Authors:** Sonia Luque Peralta, André Lindemann Dutra, Sávio Bisinoto de Leles, Juliana Silva Ribeiro, Fabricio Aulo Ogliari, Evandro Piva, Rafael Guerra Lund

**Affiliations:** 1 Faculdade Metropolitana da Grande Fortaleza, Programa de Graduação em Odontologia, Fortaleza, Ceará, Brasil; 2 Universidade Federal de Pelotas, Faculdade de Odontologia, Programa de Pós-Graduação em Odontologia (PPGO), Pelotas, Rio Grande do Sul, Brasil; 3 Universidade Federal de Pelotas, Faculdade de Odontologia, Pelotas, Rio Grande do Sul, Brasil; 4 Universidade Federal de Pelotas, Centro de Desenvolvimento Tecnológico, Pelotas, Rio Grande do Sul, Brasil

**Keywords:** Temporary dental restoration, Zinc, Microbiology, Dental leakage

## Abstract

**Objectives:**

This study investigated the physical and mechanical properties, antibacterial effect and biocompatibility of novel elastomeric temporary resin-based filling materials (TFMs) containing zinc methacrylate (ZM).

**Material and Methods:**

Experimental TFMs were prepared by mixing the zinc methacrylate with monomer, co-monomer, photoinitiator and fillers. A ZM concentration of 0 (control), 0.5% (Z0.5); 1% (Z1), 2% (Z2), or 5% (ZM5) wt% was added to the TFMs. Fermit-N (F) was used for comparison with the experimental material. Microleakage, water sorption/solubility, degree of conversion, depth of cure, ultimate tensile strength, and hardness were determined and compared. A modified direct contact test (DCT) with *Enterococcus faecalis* and a *Streptococcus mutans’* biofilm accumulation assay was carried out to evaluate the antimicrobial effect and cytotoxicity of the assay. Statistical comparisons were performed (α=5%).

**Results:**

The results showed that the physical and mechanical properties of the experimental TFMs with ZM are comparable with the properties of the commercial reference and some properties were improved, such as lower microleakage and water sorption, and higher ultimate tensile strength values. TFMs with ZM killed *E. faecalis* only after 1 h. Biofilm development of *S. mutans* was not affected by the inclusion of ZM in the experimental TFMs.

**Conclusions:**

The present findings suggest that the physical, mechanical and biological properties of the experimental TFMs with ZM are comparable with the properties of the commercial reference. However, some properties were improved, such as lower microleakage and water sorption, and higher ultimate tensile strength values.

## Introduction

Temporary filling materials (TFMs) are commonly used for coronal sealing to prevent contamination and penetration of saliva, and for microorganism products during and after endodontic treatment. Coronal microleakage appears to be of equal or greater clinical relevance as a factor in endodontic failure than apical leakage, due to risk of recontamination. ^1^ ^,^ ^2^ The literature has reported presence of residual flora after this use. ^3^ ^-^ ^5^ Therefore, dental materials are expected to have low cytotoxicity and to prevent bacterial colonization and biofilm formation on their surfaces. ^6^

Several TFMs are available commercially, such as: reinforced zinc-oxide eugenol-based; calcium sulfate-based; composite resin-based; resin reinforced glass-ionomer; and traditional glass-ionomer materials. ^1^ ^,^ ^7^ However, all these materials have been shown to adequate if placed in a thickness of 3 mm or greater. ^8^ New resin-based materials were introduced as temporary restorative materials. ^9^ ^,^ ^10^ These materials do not provide an immediately effective seal once they undergo polymerization shrinkage of 1 to 3%. ^11^ ^,^ ^12^ This contraction is compensated by the fact that they swell by absorbing water. Despite this, these materials provide good initial antimicrobial properties. ^12^

Zinc methacrylate, ^13^ a monomer that has demonstrated matrix metalloproteinase 2 (MMP-2) inhibition, ^14^ also contains a functional methacrylate group in its structure that is found in other monomers. The use of a copolymerizable monomer with zinc in its constitution is very promising. Zinc is a metallic chemical element with an antibacterial effect, ^13^ however, by the time this study was conducted, in the author's knowledge it has not been added to temporary resin-based materials.

The aim of this study was to evaluate the selective physical and mechanical properties, as well as antibacterial effects of novel temporary elastomeric filling materials containing zinc methacrylate monomer (ZMM). The null hypotheses tested were that temporary filling materials containing zinc methacrylate monomer would show antibacterial effect and physical-mechanical properties compared with the commercial reference tested.

## Materials and methods

### Material and reagents

The exothane 8 (Esstech Inc.; Essington, PA, USA); ethoxylated bisphenol A diglycidyl ether dimethacrylate with 30 ethylene oxide units (Esstech Inc.; Essington, PA, USA); triethylene glycol dimethacrylate (TEGDMA, Esstech Inc.; Essington, PA, USA), were used as received. The photosensitizer camphorquinone (CQ - Esstech Inc.; Essington, PA, USA) and the co-initiator ethyl 4-(dimethylamino) benzoate (EDAB - Sigma Aldrich; Saint Louis, Missouri, USA), diphenyliodonium hexafluorophosphate (DPIHFP) were used to render the materials light polymerizable; and as filler, silica particles and zinc methacrylate (Aldrich Chemical Co.; Milwaukee, WI, USA).

The concentrations of zinc methacrylate tested were: 0.5% (Z0.5); 1% (Z1), 2% (Z2), and 5% (Z5) by weight. A formulation without zinc methacrylate was used as experimental control (C). The Fermit-N (Ivoclar Vivadent; Schaan, Liechtenstein) (F) was used as a commercial reference. Light activation procedures were carried out using a light-emitting diode unit (Radii-cal; SDI, Bayswater, Victoria, Australia) with 1200-mW/cm^2^ irradiance and 440-480 nm of light wavelength. The radiance used for light activation of the tested materials was checked by MARC Resin Calibrator system (BlueLight Analytics; Halifax, NS, Canada).

### Microleakage

Sixty recently extracted bovine incisors were used. Cavities were prepared on the buccal surface of each tooth. The saucer-shaped cavities, measuring 3 mm in diameter and 2 mm deep were located in the middle-part of the buccal surface. Teeth were randomly divided into seven groups (n=10). After the preparations, cavities were filled with the material; covered with polyester trip and polymerized for 20 s. All materials were used according to manufacturer's instructions. The light polymerized materials were light activated. Root apexes were sealed with a chemically cured epoxy resin (Durepoxi^®^; Alba Química Indústria e Comércio Ltda., São Paulo, SP, Brazil). Two coats of nail varnish were applied on the tooth surfaces, except for the restorations, with a distance of 2 mm around their margins. The samples were stored in blue methylene solution 0.5% pH 3 - the solution of blue methylene having been buffered with sodium hydroxide until reaching pH 7 - at 23°C for 24 h, and subjected to thermal cycling in distilled water for 1000 cycles at temperatures from 5° to 55°C, with a dwell time of 30 s. The specimens were sectioned longitudinally in the buccal-lingual plane to obtain 2 slices (7 mm thick) that would be used to assess the dye infiltration. Dye leakage was evaluated by one calibrated and blinded examiner using a stereomicroscope (Tecnival, Biosystems Ltda.; Curitiba, PR, Brazil) at 40X magnification with an accuracy of 0.1 mm. The length of dye penetration was evaluated using the application ImageJ [version 1.41o, Java 1.6.0_10 (Wayen Rasband, U.S. National Institutes of Health; Bethesda, MD, USA; website: http://rsb.info.nih.gov/ij/download.html )].

### Water sorption and solubility

Ten disk-shaped (6.0 mm in diameter and 1 mm thick) specimens *per* group (n = 10) were prepared in a stainless-steel mold. The specimens were light activated for 20 s on each side; then transferred to a desiccator at 37°C and their mass monitored daily until a constant mass ( *m_1_* ) was obtained. The discs were immersed in 1.5 mL of distilled water at 37°C for 7 days, removed, blotted dry, and re-weighed (m_2_). Thereafter, the specimens were again dried in a desiccator and weighed daily to record a third constant mass ( *m_3_* ). A metallic sample holder was used in order to ensure that the samples were suspended. For each disc, water sorption ( *WS* ) and solubility ( *SL* ) data were calculated as the percentage in mass gained or lost during the sorption and desorption cycles. ^15^

### Degree of conversion

The degree of double bond conversion (GC) was evaluated by means of Fourier transform infrared spectroscopy (Prestige-21, Shimadzu, Ltd.; Tokyo, Japan), equipped with an attenuated total reflectance (ATR) device, composed of a diamond crystal with 45° mirror angle (Pike Technologies; Madison, WI, USA). Samples (n = 3) of the materials were placed on the diamond cell window of the ATR unit. A spectrum was captured before and after the polymerization process. Degree of conversion was calculated considering the vibration intensity of the elongation type of the aliphatic carbon-carbon double bond in a frequency of 1635 cm-1. A peak of 1610 cm-1 corresponding to the aromatic ring was used as an internal standard. The degree of double bond conversion versus polymerization reaction time data was plotted and Hill's three-parameter nonlinear regression was performed for curve fitting.

### Depth of cure

Depth of cure was analyzed by the scraping method. The materials (n=3) were put into a cylindrical silicone mold (6 mm diameter, 20 mm height) and irradiated with LED light curing unit through a polyester strip for 20 s. At the end of the irradiation period, the composite sample was removed from the mold and the unpolymerized material was removed from the bottom of the sample by scraping it away manually with a spatula. ^16^ Using a digital caliper, three samples of each material were used to determine the material mean depth of cure.

### Dimensional alterations

The TFMs were placed in cylindrical silicone molds (20 mm high, 6 mm in diameter) and covered with polyester strips, polymerized with a with LED light curing unit for 20 s (n=8). The samples were then removed from the molds; their length was measured with a caliper, and then they were stored in 100% humidity and incubated at 37°C. The other procedures were the same as those used in the study by Versiani, et al. ^17^ (2006) to obtain the dimensional changes of the sample after 30 days of storage.

### Ultimate Tensile Strength (UTS)

Ten dumbbell-shaped specimens (length 10 mm × width 5 mm × constriction 1 mm) were prepared for each group by using elastomer molds (n = 10). The top and bottom surfaces were light-activated for 20 s. After fabrication, the tensile test was performed in a mechanical testing machine (DL500; EMIC, São José dos Pinhais, PR, Brazil) at a crosshead speed of 1 mm/ min until failure. UTS values were calculated in MPa.

### Shore hardness measurements

Measurements were made according to the ASTM D2240 by using the Shore D scale hardness tester (PanTec, Panambra Ind. e Técnica SA, São Paulo, SP, Brazil). Measurements were also made on specimens approximately 15 mm thick and 6 mm diameter (n=4), with 100 g load *per* 10 s at each indentation. However, measurements were still made at the specified depths of 0.5; 5; 10 and 13 mm. Three readouts were taken for each depth in the specimens and the mean value calculated.

### Biofilm accumulation test

*Streptococcus mutans* UA159 - a well-described cariogenic pathogen and a strain selected for genomic sequencing ^18^ - was cultured overnight at 37°C in Brain Heart Infusion broth (BHI) in an anaerobic atmosphere. The optical density (OD) at 600 nm of the bacterial suspension was adjusted to 0.5.

Specimens measuring 6 mm in diameter and 1 mm (n=8) thick were suspended in the wells of a 24-well plate. Within each well, 2-mL of ultrafiltered 10 kDa molecular weight cut-off membrane (Amicon; Danvers, Mass., USA) tryptone-yeast extract broth (UTYEB) supplemented with 1% sucrose and 20 μL of bacterial suspension were inoculated. For 3 days, the biofilms on discs were washed 3 times in 0.9% NaCl and transferred to a new plate with fresh UTYEB containing 1% sucrose, for 24 h at 37°C in an environment of 5-10% CO_2_ in anaerobic jars (Anaerobac; Probac do Brasil Produtos Bacteriológicos Ltda.; Santa Cecília, SP, Brazil). After 72 h of biofilm growth, the discs were washed in 0.9% NaCl and individually transferred to tubes containing 1 mL of 0.9% NaCl. The tubes were sonicated at 30W in a probe ultrasonicator, model DES500 (Unique Group, Indaiatuba, SP, Brazil) for 30 s to detach the biofilms formed. An aliquot of 100 μl of the suspension was serially diluted, and 20 μl of each dilution were inoculated on BHI agar (Difco, Becton Dickinson and Co.; Sparks, MD, USA) to determine the number of viable microorganisms. ^19^ The plates were incubated at 37°C for 72 h, with 5-10% CO_2_. The results were expressed as CFU/mg of biofilm dry weight. ^20^

### Modified direct contact test

*Enterococcus faecalis* ATTC4083 was cultured overnight at 37°C in tryptic soy agar (TSA) plates in an aerobic atmosphere. *E. faecalis* was suspended in tryptic soy broth (TSB) and adjusted to an optical density (OD) of 0.5 at 600 nm.

Cylinders measuring 6 mm in diameter and 1 mm thick were placed in the wells of a 96-well plate. 10 μL of bacterial suspension was placed on the surface of the materials tested. Strain suspensions (10 μL) placed in uncoated wells served as nonexposed (positive) control. All samples were incubated aerobically at 37°C in >95% humidity for 1 and 24 h; then 240 mL of TSB was added to each well and gently mixed with a pipette for 1 min. Serial dilutions were prepared in TSB and plated onto TSA. After aerobic incubation at 37°C for 24 h, CFU/mL were calculated. ^21^ Experiments were performed in duplicate.

### Cytotoxicity assay

Discs of each TFM were sterile in cylindrical silicone discs measuring 5 mm in diameter and 1 mm high. Cytotoxicity of the TFMs was assessed after 24 h. Control samples containing only culture medium were treated similarly and undiluted extracts were used for the testing. The viability of fibroblast (NCTC clone 929) cells was determined by measuring the reduction of soluble MTT (3-(4,5-dimethylthiazol-2- yl)-2, 5-diphenyltetrazolidium bromide - Sigma; St. Louis, MO, USA) to water-insoluble formazan. Cells were seeded at a density of 2×10 ^4^ cell *per* well at a volume of 200 μl in 96-well plates and grown at 37°C in an atmosphere of 5% CO_2_ 95% for 24 h. The medium was aspirated and replaced with 200 μL/well extract or control medium, and incubated for 24 h. The medium was removed, 180 mL of medium and 20 mL MTT were added to each well and they were incubated for 4 h. DMSO was added to each well, and was solubilized on a shaker for 5 min. The formazan content of each well was computed as a percentage of the control group (untreated cells). Experiments were performed in triplicate. Cytotoxicity responses were rated as severe (30%), moderate (30%-60%), slight (60%-90%), or noncytotoxic (>90%). ^22^

### Statistical analyses

For statistical analysis, the Sigma Stat^®^ for Windows Software®, Version 3.5 (Systat Software, Inc.; Point Richmond, CA, USA), configured with a pre-set alpha of 0.05 was used. The data were analyzed to verify normal distribution and variance homogeneity. Data were analyzed using One-Way ANOVA, Two-way ANOVA, Tukey tests and the Fisher's least significance difference (LSD) *post hoc* test for pair-wise comparisons of means. For all cases, the level of significance was set at (α<0.05).

## Results

**Figure 1:**
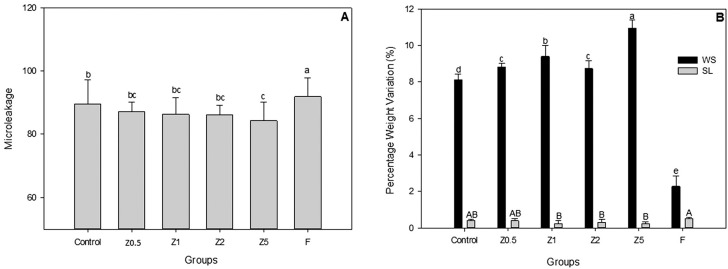
(A) Microleakage means of experimental TFMs with ZM and the commercial reference; (B) Water sorption and Solubility expressed in %wt

The microleakage means recorded for the experimental groups were analyzed statistically by Kruskal-Wallis. A difference in microleakage means of experimental TFMs with ZM and the commercial reference were observed. The percentage of mean and standard deviations are shown in Figure 1A . Z5 had lower score than the control and the commercial (F^®^) (P<0.005), but it did not differ from Z0.5, Z1 and Z2.

The TFMs containing ZM in their formulations showed statistically higher water sorption values than Group F and control (p<0.001) ( Figure 1B ). The TFM with highest concentration of ZM (Z5) absorbed the most water.

For solubility ( Figure 1B ), the experimental groups Z1, Z2, Z5 presented less solubility than Group F^®^ (p<0.05), with statistic difference observed between them (p<0.001). Table 1 shows the degree of conversion, depth of cure, dimensional alterations and cohesive strength of the TFMs. The percentage of degree of conversion (DC%) from TFMs with ZM were significantly higher than that of Group F (p<0.034)( Table 1 ). The depth of cure ranged between 7.43±0.4 mm for F^®^ and 15.43±0.1 mm for TFM with ZM. Numerically higher depth of cure could be observed in Z1 and Z2. However, all TFMs with ZM were statistically dissimilar (p<0.002).

**Table 1:** Physical and mechanical properties of the different materials tested (Mean±SD)

	Degree of conversion (%)	Depth of cure (mm)	Dimensional alterations (%)	Cohesive strength (MPa)
Control	96.46±2.8^a^	15.23±0.1^ab^	2.95±0.1^a^	6.69±1.6^c^
Z0.5	97.21±1.8^a^	15.32±0.2^ab^	3.10±0.8^a^	8.16±1.4^c^
Z1	97.33±1.1^a^	15.43±0.1^a^	2.33±0.1^a^	10.5±2.3^b^
Z2	98.56±0.8^a^	15.41±0.1^a^	2.90±0.5^a^	13.2±2.3^a^
Z5	97.55±0.8^a^	14.86±0.6^a^	3.31±1.2^a^	13.9±1.9^a^
F	27.59±6.7^b^	7.43±0.4^b^	1.07±0.6^b^	9.58±1.9^b^

Different lowercase letters within each column indicate statistically significance (p<0.05)

There were no statistically significant differences in dimensional changes between experimental TFMs tested, however Group F showed statistically fewer dimensional changes (p=0.016). The addition of ZM also resulted in improved mean cohesive strength values of the TFMs ( Table 1 ). Groups Z2 and Z5 showed the highest UTS values (p<0.001).

Table 2 showed the shore hardness of TFMs at different depths. There were no significant differences in the hardness of the specimens assessed (p=0.878). The results revealed that the mean hardness values were similar for different depths of cure ( *p* =0.080), and there was no statistically significant interaction between specimens and depth of cure (P=0.402). Group F was excluded from calculations because of the pattern of absent data.

**Table 2:** Hardness (Shore D Units) of TFMs with ZM at different cure depths (Mean±SD)

	Control	Z0.5	Z1	Z2	Z5	F
0.5 mm	46.50±2.1	45.17±2.2	48.67±2.4	48.50±2.4	48.00±1.7	47.92±3.2
5 mm	45.25±1.4	46.83±2.8	46.75±2.7	49.00±2.6	48.67±1.1	45.33±2.7
10 mm	45.92±1.2	46.58±1.7	47.92±1.5	50.08±1.2	49.08±2.0	*
13 mm	42.00±1.9	44.25±2.9	47.83±2.9	49.08±2.3	47.17±4.3	*

Different letters indicate statistical difference (p<0.05).

* No depth of cure was possible

**Figure 2:**
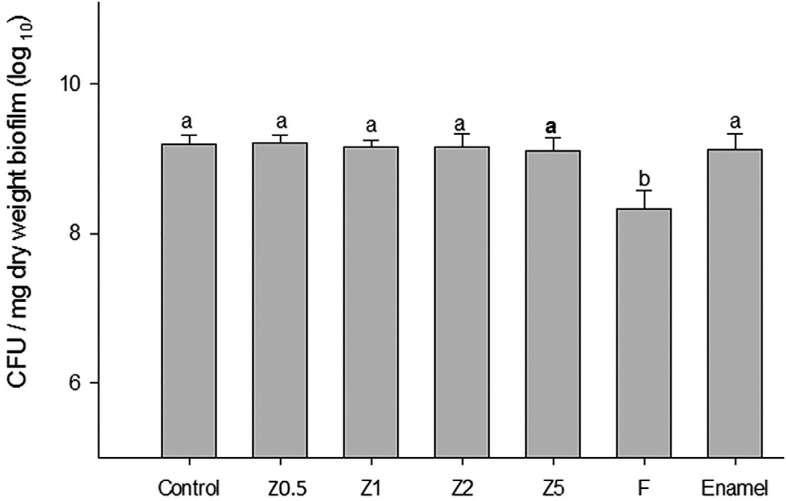
Test of *S. mutans* UA159 accumulation after 3 days of biofilm formation under conditions exposure to 1% sucrose

**Figure 3:**
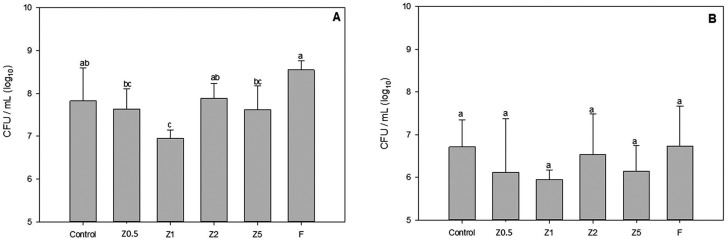
Survival of *E. faecalis* ATCC4083 after direct contact with temporary filing materials (A) for 1 h and (B) for 24 h

In the *S. mutans* UA159 biofilm accumulation test ( Figure 2 ), Group F showed lower CFU/mg than all the other experimental TFMs (P<0.001). With regard to the direct contact test after 1 h ( Figure 3A ), Group F showed lower antibacterial effect than the other groups (p<0.001). Z0.5 and Z5 were similar to control and Z2, and Z1 was the group that showed the highest bacteria reduction after 1 h, not being, however, statistically significant (p>0.05). After the 24 h in direct contact test, all TFMs with ZM were statistically similar (p=0.058).

**Figure 4:**
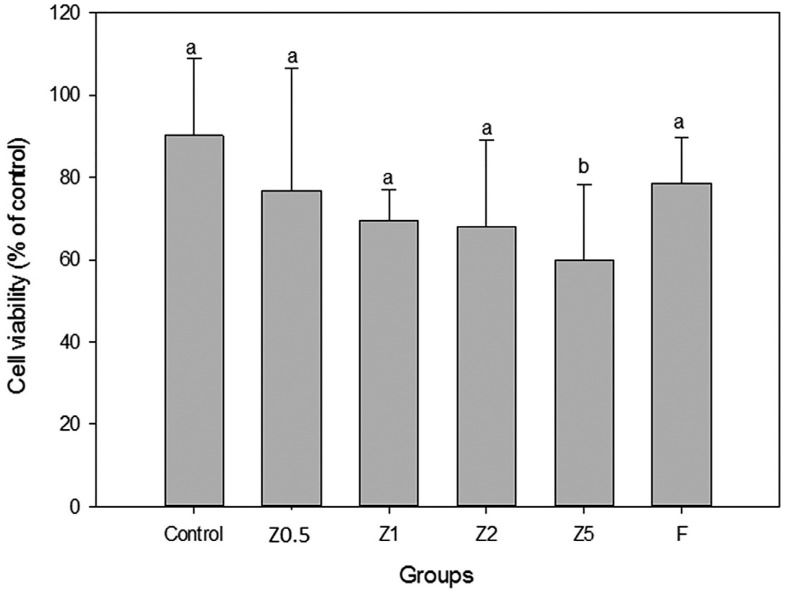
Cytotoxic effects on 929 fibroblast cells after exposure to TFMs eluate

The results of the MTT assay are presented in Figure 4 . According to statistical analysis, only Z5 were shown to present higher cytotoxicity after 24 h of exposre in DMEM (p = 0.044).

## Discussion

Temporary resin-based materials should have satisfactory mechanical properties to be accepted as a successful temporary material. The results obtained in this investigation indicated that TFMs with ZM had suitable mechanical properties that were at the same level as, or superior to, the properties of a commercial reference (F), depending on ZM concentration. The incorporation of ZM improved some properties such as ultimate tensile strength, water sorption and microleakage. The null hypothesis was partially accepted, since the experimental material containing zinc methacrylate provided antibacterial effect similar to that of the control in the DCT assay.

Z5 was shown to have the least microleakage, and its properties may be effective for sealing the edges of the tooth. Furthermore, Z5 showed the largest amount of water sorption and least microleakage. This is explained because the TFMs undergo polymerization shrinkage and, after contact with water, they swell. Moreover, the higher sorption of experimental TFMs with ZM was most likely due to the presence of Zn+ ^2^ , which form a network of Zn bonds and ZnOH in the polymer chains of the resin-based temporary material in the presence of water. In a more heterogeneous network, the space created between the polymer clusters (microporous) is larger and accommodates a higher amount of water. ^15^

In the DC test of the TFMs, no significant difference was observed between the control and the other groups containing ZM. The incorporation of ZM did not affect the DC, and these results are contrary to other studies, ^13^ probably due to the differences in composition of the materials assessed in these studies. In the present study, the authors used exothane 8^®^. This component showed almost full polymerization, and according to the literature, the higher the degree of conversion, the higher the material stability in the face of hydrolysis degradation phenomena. ^23^ This will likely generate a long-lasting material with improved physical-mechanical properties.

The depth of cure depends on different factors, including: irradiation energy, wavelength, and light penetration into the materials. The amount and size of filler particles also affect this phenomenon. Incorporation of ZM into the materials showed no changes in depth of cure, probably because these materials have transparency. Moreover, these results differed from other studies, ^13^ ^,^ ^16^ probably because, in those studies, larger amounts of zinc methacrylate and of zinc oxide nanofillers were incorporated. Their results showed a decrease of nearly half in the depth of cure. ^16^

The hardness was evaluated at different cure depths and showed similar results of 0.5 to 13 mm of curing depth. The experimental materials showed that, even when providing a greater depth of cure 10 mm, they retained their hardness. The UTS increased in experimental TFMs, and this may be explained by the higher degree conversion that improves some mechanical properties. ^24^

Dimensional changes of experimental TFMs during and after setting are a source of concern to clinicians. The dimensional stability of resin-based materials can be affected by polymerization shrinkage, thermal contraction, expansion, and interaction with an aqueous oral environment. ^15^ Clinically, the expansion of a filling material in cavities may increase the application of lateral force and lead to crown and root-crown fractures. In addition, it is recommended to consider analyzing, as a future study, their effect on real human teeth subjected to fatigue, as well as analyze the mechanical performance of the tooth/ provisional material complex in order to explain the relation between polymerization shrinkage and whether a high expansion can cause damage on a weakened tooth structure.

The release of oxygen and zinc ions may provide the ZnO with an antibacterial effect. The possible antimicrobial mechanism of ZnO occurs via the leaching of Zn ^2^ + into the microbiological medium, ^25^ inhibiting the active transport and metabolism of sugars, as well as disrupting enzyme systems of dental biofilms by exchanging magnesium ions essential for the enzymatic activity of plaque. ^26^ Zinc can also reduce the acid production of *S. mutans* biofilms due to its ability to inhibit glucosyl transferase activity. ^27^ ^,^ ^28^

A point to consider is the fact that this is the first study that has evaluated zinc methacrylate in models of *S. mutans* biofilm. It has already been indicated in the literature that biofilms are organized in communities of microorganisms coated with extracellular polysaccharides and are more resistant than bacteria without that organization. This could explain the effect of (10-30 wt%) zinc methacrylate on a biofilm model with *S. mutans* UA159. ^13^

The DCT is a test for evaluating solid materials that have components with low solubility. In the DCT method, bacteria are under a previously controlled direct contact with the desired material, and after this, bacterial growth can be quantified. ^21^ In this study, the authors assessed two-timed intervals of TFMS exposure to *E. faecalis*: periods of 1 and 24 h. The results provided on Figure 3A showed that TFMs can exert antibacterial effect against *E. faecalis* after 1 h of exposure at the concentrations of Z0.5, Z1 and Z5 tested. These results could not be compared with those of other studies because zinc was a functionality of methacrylate. After 24 h, all groups were similar, probably because of the antimicrobial effect of the zinc components of TFMs, or because there was a higher bacterial growth that resulted in zinc ion saturation in the media, and lack of nutrients causing the death of bacteria in the different groups tested. The antibacterial effects of Zn are known, ^25^ however, attempting to explain the antimicrobial mechanism of ZM only by the leaching of Zn^2+^ cannot be expected with this study, probably due to the difficulty of releasing the Zn ion, which can be supported by the data regarding the degree of conversion and solubility. However, a considerable antibacterial activity of ZnO has also been attributed to their biological effect to the generation of reactive oxygen species on the surface of these oxides. The advantage of using this inorganic oxide as antimicrobial agent is that they contain mineral elements essential to humans and exhibit strong activity even when administered in small amounts. This activity is quantitatively evaluated by studying the growth medium caused by the bacterial metabolism. In this case, the cell wall rupture of bacteria must be due to the surface activity of ZnO in contact with the bacteria. ^29^

For the cytotoxicity test, the Z5 showed lower cell viability than all the other groups. This could be explained by the higher concentration of ZM. However, in this study, functionalized zinc was used. The results showed that the control material (ZM-free) had no cytotoxic effect on cells in the MTT test.

## Conclusions

These novel composites based on zinc methacrylate exhibited promising elastomeric features and better sealing capacity, likely provided by increased water sorption as a compensatory feature with regard to composite shrinkage. In addition, in the direct contact test (DCT), the test TMF composites showed biocompatibility and an antibacterial effect against *E. faecalis.*
